# Triggering of Transmural Infarctions, but Not Nontransmural Infarctions, by Ambient Fine Particles

**DOI:** 10.1289/ehp.0901624

**Published:** 2010-04-30

**Authors:** David Q. Rich, Howard M. Kipen, Junfeng Zhang, Leena Kamat, Alan C. Wilson, John B. Kostis, for the Myocardial Infarction Data Acquisition System Study Group (MIDAS 12)

**Affiliations:** 1 School of Public Health, University of Medicine and Dentistry of New Jersey, Piscataway, New Jersey, USA; 2 Environmental and Occupational Health Sciences Institute, Robert Wood Johnson Medical School and Rutgers University, Piscataway, New Jersey, USA; 3 Robert Wood Johnson Medical School, University of Medicine and Dentistry of New Jersey, Piscataway, New Jersey, USA

**Keywords:** air pollution, epidemiology, myocardial infarction

## Abstract

**Background:**

Previous studies have reported increased risk of myocardial infarction (MI) after increases in ambient particulate matter (PM) air pollution concentrations in the hours and days before MI onset.

**Objectives:**

We hypothesized that acute increases in fine PM with aerodynamic diameter ≤ 2.5 μm (PM_2.5_) may be associated with increased risk of MI and that chronic obstructive pulmonary disease (COPD) and diabetes may increase susceptibility to PM_2.5_. We also explored whether both transmural and nontransmural infarctions were acutely associated with ambient PM_2.5_ concentrations.

**Methods:**

We studied all hospital admissions from 2004 through 2006 for first acute MI of adult residents of New Jersey who lived within 10 km of a PM_2.5_ monitoring site (*n* = 5,864), as well as ambient measurements of PM_2.5_, nitrogen dioxide, sulfur dioxide, carbon monoxide, and ozone.

**Results:**

Using a time-stratified case-crossover design and conditional logistic regression showed that each interquartile-range increase in PM_2.5_ concentration (10.8 μg/m^3^) in the 24 hr before arriving at the emergency department for MI was not associated with an increased risk of MI overall but was associated with an increased risk of a transmural infarction. We found no association between the same increase in PM_2.5_ and risk of a nontransmural infarction. Further, subjects with COPD appeared to be particularly susceptible, but those with diabetes were not.

**Conclusions:**

This PM–transmural infarction association is consistent with earlier studies of PM and MI. The lack of association with nontransmural infarction suggests that future studies that investigate the triggering of MI by ambient PM_2.5_ concentrations should be stratified by infarction type.

Most previous studies (D’Ippoliti et al. 2003; Peters et al. 2001, 2005; Pope et al. 2006; Zanobetti and Schwartz 2005), but not all (Sullivan et al. 2005), that have investigated the triggering of myocardial infarction (MI) by particulate matter (PM) air pollution concentrations in the hours and days before MI onset have reported an association. Other studies have reported increased mortality due to MI or increased mortality or cardiovascular admissions among MI survivors associated with increases in PM over the previous few days (Braga et al. 2001; von Klot et al. 2005; Zanobetti and Schwartz 2007). Several studies have investigated whether certain subgroups are particularly susceptible and have reported increased cardiovascular effects among those with diabetes (Dubowsky et al. 2006; Goldberg et al. 2001; Liu et al. 2007; O’Neill et al. 2005; Zanobetti and Schwartz 2002) and among patients with chronic obstructive pulmonary disease (COPD) (Naess et al. 2007; Zanobetti and Schwartz 2005; Zanobetti et al. 2000). However, Zanobetti and Schwartz (2005) reported increased susceptibility among patients with COPD but not among persons with diabetes.

Numerous researchers have reported that the percentage of infarctions that are nontransmural has been increasing (Goff et al. 2000; Hellermann et al. 2003; Kostis et al. 2007; Myerson et al. 2009; Roger et al. 2006, 2010; Rogers et al. 2008). More recently, Shao (2008) noted a similar secular trend in clinical presentation of MI to emergency departments (EDs) in New Jersey. In short, 72% of persons admitted to hospitals in New Jersey from 1990 through 1992 for MI had had transmural infarctions, and only 28% had had nontransmural infarctions. Since then, however, this pattern has reversed. From 2002 through 2004, most of the admissions for MI were for nontransmural infarctions (63%), with only 37% for transmural infarctions (Shao 2008). These changes may be due in part to improvements in preventive pharmacotherapies (statins, beta blockers, aspirin), interventional procedures [angioplasty, coronary artery bypass graft (CABG)], more sensitive diagnostic tests (troponins), and treatment of the MI upon ED arrival (reperfusion therapy, increased use of antiplatelet agents). It has not been reported whether PM with aerodynamic diameter ≤ 2.5 μm (PM_2.5_) triggers all infarctions, whether PM–MI associations differ in magnitude, or whether these associations are restricted to PM–transmural or PM–nontransmural infarctions alone. Such investigations may provide insight into the mechanisms by which PM may trigger cardiovascular events.

Using the same data as Shao (2008), we attempted to replicate, in New Jersey, previous MI–PM_2.5_ studies that were conducted in other U.S. and European cities. We hypothesized that increases in mean PM_2.5_ concentration on the same day and a few days before ED arrival for MI may be associated with increased risk of MI and that these effect estimates would be greater for persons with diabetes and those with COPD than for individuals without these conditions. Further, we explored whether there were differences in risks associated with increases in ambient PM_2.5_ concentration between transmural and nontransmural infarctions.

## Materials and Methods

### Study population and outcome definition

Using the Myocardial Infarction Data Acquisition System (MIDAS), a statewide surveillance system in New Jersey that combines hospital discharge data and death certificate registration data (Kostis et al. 1994, 2001), we extracted all records with a primary diagnosis of acute MI [*International Classification of Diseases*, version 9 (ICD-9), codes 410.01, 410.11, 410.21, 410.31, 410.41, 410.51, 410.61, 410.71, 410.81, and 410.91] for patients who were admitted between 1 January 2004 and 31 December 2006, were ≥ 18 years of age, were residents of New Jersey at the time of their MI, and who were without a previous diagnosis of MI (ICD-9 code 412). Because the “1” in the fifth digit of the ICD-9 code (e.g., 410.01) indicates a first MI for the subject, we also used this designation to exclude those persons who had had a previous MI. Those who were not admitted into the hospital (e.g., patient declined admission, patient died before admission) were not included in this data set. We classified patients with an MI coded as 410.7 (subendocardial infarction) as having a nontransmural infarction, and those with codes 410.0 (infarction of anterolateral wall), 410.1 (infarction of other anterior wall), 410.2 (infarction of inferolateral wall), 410.3 (infarction of inferoposterior wall), 410.4 (infarction of other inferior wall), 410.5 (infarction of other lateral wall), and 410.6 (true posterior wall infarction) as having transmural infarctions.

From these extracted data, we also retained the following variables: date and hour of ED admission; ZIP code of residence; age; sex; race; variables indicating the presence or absence of other comorbid conditions (based on ICD-9 codes)—congestive heart failure (ICD-9 code 428.0), arrhythmia (ICD-9 codes 426.0, 426.10, 426.12, 426.2, 426.3, 426.4, 426.5, 426.6, 426.89, 426.9, and 427), hypertension (ICD-9 codes 401–405), diabetes (ICD-9 code 250), COPD (ICD-9 codes 490–496), and history of ischemic heart disease (ICD-9 codes 410–414); and variables indicating in-hospital and postinfarction procedures (based on ICD-9 code)—angioplasty, CABG, and catheterization.

This study and the original MIDAS study were approved by the University of Medicine and Dentistry of New Jersey–New Brunswick Institutional Review Board. MIDAS was also approved by the New Jersey Department of Health and Senior Services Institutional Review Board.

### Air pollution

Using ambient pollutant measurements from the New Jersey Department of Environmental Protection and from U.S. Environmental Protection Agency (EPA) Web sites (U.S. EPA 2008), we used hourly concentrations of PM_2.5_ (7 monitoring stations), nitrogen dioxide (NO_2_; 9 stations), sulfur dioxide (SO_2_; 14 stations), carbon monoxide (CO; 13 stations), and ozone (O_3_; 15 stations) for the study period 1 January 2004 to 31 December 2006. For each patient, we calculated the distance between each PM_2.5_ monitor (in operation at the time of the MI) and the patient’s residence and assigned PM_2.5_ measurements from the closest monitor to their residence. Those patients who lived > 10 km from a PM_2.5_ monitoring station were excluded from PM_2.5_ analyses. We calculated mean PM_2.5_ concentrations for each successive 24-hr period before ED arrival for the MI (e.g., mean of hours 0–23 before ED arrival; 24–47 mean of hours before ED arrival, etc.). If > 6 hr (> 25%) of a 24-hr period of PM_2.5_ concentrations were missing, we set the mean for this 24-hr period of PM_2.5_ concentration to missing. We used these mean concentrations in all analyses. We repeated this monitor matching and mean concentration calculation process for each of the other pollutants.

### Weather

Hourly temperature and dew point measurements were made at the Newark, Caldwell, Somerset, and Trenton, New Jersey, airports during the study period. We used the airport monitor closest to each patient’s residence to provide the weather observations for that patient during the study period. After calculating 24-hr mean temperature and dew point, in the same manner as for the pollutant concentrations, we calculated 24-hr mean apparent temperature (Steadman 1979; Zanobetti and Schwartz 2005) as a measure of each patient’s perceived air temperature given the humidity and used these values in all analyses.

### Study design

We used a time-stratified case-crossover design (Levy et al. 2001; Maclure 1991) that has previously been used in studies of ambient air pollution and MI (D’Ippoliti et al. 2003; Pope et al. 2006; Sullivan J et al. 2005; Zanobetti and Schwartz 2005), as well as other cardiovascular outcomes (Rich et al. 2005, 2006a, 2006b; Wellenius et al. 2005). In this design, each patient contributed information as a case during the period immediately before the MI and as a matched control during times when an MI did not occur. The case-crossover design is analogous to a matched case–control study, but instead of estimating the relative risk of MI comparing exposure between persons (i.e., cases vs. controls), we estimated the relative risk of MI comparing exposure during different time periods within the follow-up time of each case of MI. Because case periods and their matched control periods are derived from the same person and a conditional analysis is conducted, non-time–varying confounders such as age, comorbidities, and history of long-term smoking were controlled by the study design. However, variables that may be related to both air pollution and the incidence of MI that vary over short time periods, such as weather conditions, which vary day to day, were possible confounders and were included in our analytic models. Case periods were defined as the 24-hr period before ED admission for MI, whereas control periods (three or four per case depending on the number of days in the calendar month) were matched to the case period by day of the week, time of the day, year, and month. For example, if a person arrived at the ED with an MI at exactly 0000 hours on 20 March 2009, then the 24-hr case period was the prior 24 hr (0000 hours to 2359 hours on Thursday 19 March), and the control periods were 0000 hours to 2359 hours on the three previous Thursdays (5, 12, and 26 March 2009). Pollutant concentrations corresponding to these case and control periods were contrasted in all analyses.

### Main analysis

Using a conditional logistic regression model stratified on each MI (one case and three or four control periods), we regressed case–control status (i.e., case period = 1, control period = 0) against the mean PM_2.5_ concentration in the 24 hr before ED arrival. Using Akaike’s information criterion to select the optimal lag time and number of degrees of freedom for apparent temperature, we also included a natural spline (3 degrees of freedom) of the mean apparent temperature in the 48 hr before ED arrival in the model. We then reran this same model, replacing the 24-hr mean PM_2.5_ concentration (for both case and control periods) with one of six lagged mean PM_2.5_ concentrations (i.e., mean of hours 24–47, 48–71, 72–95, 96–119, 120–143, or 144–167 before ED arrival) to estimate the risk of MI associated with each lagged PM_2.5_ concentration. From each model, we present the odds ratio (OR) and its 95% confidence interval (CI) scaled to the interquartile range (IQR) of PM_2.5_ observed during the study period. A *p*-value < 0.05 was used to indicate statistical significance.

Next, we examined whether the risk of MI associated with an IQR increase in mean PM_2.5_ concentration in the 24 hr before ED arrival was different for patients with transmural versus nontransmural infarctions. We reran the same model described above for patients with transmural infarctions only, and then for patients with nontransmural infarctions only. To examine potential effect modification by other factors, including COPD, diabetes, age (< 65 years, ≥ 65 years), sex, race (white, black, other), and season (winter = December, January, February; summer = June, July, August), we used an interaction term (e.g., COPD × PM_2.5_) in the main model.

### Sensitivity analyses

To assess the stability of our PM_2.5_ relative risk estimates after adjusting for gaseous pollutant concentrations, we created two-pollutant models (PM_2.5_ + NO_2_, PM_2.5_ + CO, PM_2.5_ + SO_2_, PM_2.5_ + O_3_) using mean pollutant concentrations from the 24 hr before ED arrival. We also evaluated whether any association between ED arrival for MI and the mean PM_2.5_ concentration in the previous 24 hr was independent of concentrations from previous 24-hr periods. We reran the same conditional logistic regression model described above, including all seven lagged PM_2.5_ concentrations (i.e., the mean PM_2.5_ concentrations from the 24 hr before ED arrival for MI, and the mean concentrations from the previous six lagged 24-hr periods).

All data sets included in the analyses were constructed using SAS software (version 9.1.3; SAS Institute Inc., Cary, NC), and all analyses were conducted using R (version 2.6.1; R Foundation for Statistical Computing, Vienna, Austria). The authors had full access to the data and take responsibility for its integrity.

## Results

During the study period, a total of 37,791 patients were admitted to nonfederal New Jersey hospitals for a first acute MI; of these, 5,864 lived within 10 km of a PM_2.5_ monitoring site and had PM_2.5_ data available for analysis (i.e., PM_2.5_ data available for at least 18 of the 24 hr before ED arrival for the index MI). Study patients were predominantly older (59% > 65 years of age), white (69%), and male (56%). Sixty-two percent had hypertension, 61% had a history of ischemic heart disease, and 30% had diabetes (Table 1). Those who were excluded from our analysis were similar to persons who were included in age (61% > 65 years of age), sex (57% male), and comorbidities (e.g., 59% with hypertension), but they were slightly more likely to be white (82%). Subjects included in the analysis who experienced a nontransmural infarction were generally older and were more likely to have had cardiorespiratory comorbidities than were those who experienced a transmural infarction. Compared with the subjects who had a nontransmural infarction, those who had a transmural infarction were more likely to have had an angioplasty and/or catheterization postinfarction but less likely to have had CABG (Table 1). Of note, there were seven monitors that made PM_2.5_ measurements in 2006 but only three in 2004 and 2005, which resulted in a larger number of patients living within 10 km of a PM_2.5_ monitoring site and thus available for analysis in 2006 (Table 2).

Of the seven monitors that were continuously measuring PM_2.5_ during the study period, patients were most often assigned to the Elizabeth Lab (32%), Camden Lab (26%), and New Brunswick (18%) monitors. The distribution of mean daily PM_2.5_ concentrations at each of these seven monitors is shown in Table 2. When combining all the monitors, PM_2.5_ concentrations had a median of 11.7 μg/m^3^; 5th and 95th percentiles of 3.6 and 31.5 μg/m^3^, respectively; and 25th and 75th percentiles of 7.4 and 18.3 μg/m^3^, respectively (IQR = 10.8 μg/m^3^). We scaled all our effect estimates presented below by this IQR. Table 3 shows Pearson correlation coefficients for individual pollutants and apparent temperature.

Next, we separately estimated the risk of ED admission for MI associated with each IQR increase in the mean PM_2.5_ concentration in the previous 24 hr, and lagged 24-hr periods (hours 24–47, 48–71, 72–95, 96–119, 120–143, and 144–167), adjusting for apparent temperature during that same lag period. Each 10.8-μg/m^3^ increase in the mean PM_2.5_ concentration in the 24 hr before ED arrival was not associated with an increased risk of MI (Table 4). Similarly, we found no associations between ED admission for overall MI and any of the lagged PM_2.5_ concentrations. However, when we then restricted our analysis to transmural infarctions only we found a significantly increased risk associated with each 10.8-μg/m^3^ increase in PM_2.5_ concentration in the previous 24 hr (OR = 1.10; 95% CI, 1.01–1.20). When examining lagged PM_2.5_ concentrations, the relative risk estimates were mostly > 1.0, but none was statistically significant. In contrast, when we restricted our analysis to nontransmural infarctions, we found no increased risks associated with any lagged PM_2.5_ concentrations (Table 4).

Because we found an association with transmural infarctions only, we restricted all further analyses to this infarction type and evaluated whether several factors modified this association. Patients with preexisting COPD and those < 65 years of age had substantially larger risks of transmural infarction associated with each 10.8 μg/m^3^ increase in PM_2.5_ concentration in the previous 24 hr than did patients without preexisting COPD and those ≥ 65 years of age (Table 5). We found no difference in PM_2.5_ relative risk estimates by race, sex, season, or whether subjects had diabetes (Table 5).

We then included mean PM_2.5_ and NO_2_ concentrations from the 24 hr before ED arrival simultaneously in a model (*n* = 1,262 patients with transmural infarctions with both PM_2.5_ and NO_2_ mean concentrations). The PM_2.5_ relative risk estimate in this two-pollutant model was not substantially different from the single-pollutant model on the same *n* = 1,262 patients (Table 6). This was also true when adjusting for CO, SO_2_, and O_3_. IQR increases in CO, SO_2_, and O_3_ were not associated with significantly increased risks of transmural infarctions in any single- or two-pollutant model (Table 6). Next, when including all seven lagged PM_2.5_ concentrations in the same model, the risk associated with the 24-hr moving-average PM_2.5_ concentration was larger and still statistically significant (OR = 1.15; 95% CI, 1.04–1.28). All the PM_2.5_ concentration relative risk estimates for the other lag periods were smaller than the 24-hr moving-average relative risk estimate, and none was statistically significant (data not shown).

## Discussion

Using a large multiyear (2004–2006) statewide data set of hospital admissions for first MI, we found no association between admission for MI overall and PM_2.5_ concentrations in the previous week. However, when we restricted the analysis to patients with transmural infarctions, we found a significant 10% increase in the risk of a transmural infarction associated with the mean PM_2.5_ concentration in the 24 hr before ED arrival. This association persisted with adjustment for gaseous pollutant concentrations in the prior 24 hr and with adjustment for PM_2.5_ concentrations during each of the previous six 24-hr periods. Further, patients with COPD, but not diabetes, were particularly susceptible to acute increases in ambient PM_2.5_ concentrations. We found no association with this same PM_2.5_ concentration and nontransmural infarctions.

Our findings are consistent with previous studies (D’Ippoliti et al. 2003; Peters et al. 2001; Peters et al. 2005; Pope et al. 2006; Zanobetti and Schwartz 2005) in indicating an acute association between PM and MI onset, although the method of estimating MI onset time (day of hospital admission for MI, time of symptom onset estimated by patient, etc.), lags of pollutant concentrations examined (lag days 0–6, 0–6 lagged 24-hr periods before ED arrival for MI), and specific particle sizes examined (PM_10_, PM_2.5_) are not uniform. Previous studies reported PM associations with all MIs, not just transmural infarctions (D’Ippoliti et al. 2003; Peters et al. 2001; Peters et al. 2005; Pope et al. 2006; Zanobetti and Schwartz 2005). However, these studies drew data from earlier time periods [e.g., Peters et al. (2001), Boston, Massachusetts, 1995–1996; Peters et al. (2005), Augsburg, Germany, 1999–2001; D’Ippoliti et al. (2003), Rome, Italy, 1995–1997; Zanobetti and Schwartz (2005), 21 U.S. cities, 1985–1999)] when most infarctions were likely transmural. Therefore, their reports of increased relative risk of MI are consistent with our finding of increased relative risk of transmural infarctions only. Thus, our study adds to the body of knowledge that relates increased risk of MI with increases in PM in the previous hours and days, but ours is the first to report that associations are restricted to transmural infarctions.

Pathophysiologic pathways proposed as mechanisms underlying previously reported PM/MI associations include systemic inflammation, endothelial dysfunction, disturbance of autonomic tone, and enhanced coagulation/thrombosis (Brook et al. 2004). Our finding of an acute (within 24 hr) association between PM and transmural infarctions, but not nontransmural infarctions, suggests another mechanism or pathway, perhaps related to those listed above, by which particles may trigger an MI. In a recent study, Bartoli et al. (2009) reported decreased myocardial blood flow in canines associated with concentrated air particles exposure, but not with filtered air exposure, after experimental occlusion of the coronary artery. Cardiac work was not increased by PM in this dog model, and data suggested that blood flow reductions were due to increased coronary vascular resistance, perhaps related to more limited recruitment of collateral vessels. Thus, particle inhalation, by reducing compensatory mechanisms, may transform limited (nontransmural) injury into more extensive (transmural) infarction. This finding is consistent with other research in which PM has been associated with increased rates of ST-segment depression in humans (Gold et al. 2005; Pekkanen et al. 2002) or in canine model markers of ischemia (Wellenius et al. 2003). There is also increasing support in the literature for the prothrombotic effects of air pollutants that indicates the possibility that limitations in revascularization after a primary plaque-related thrombotic event could be due to enhanced thrombosis in general (Delfino et al. 2009; Jacobs et al. 2010; Lucking et al. 2008). A recent study that examined the association between PM and acute coronary events demonstrated greater relative risk in those individuals with angiographically documented previous coronary disease (Pope et al. 2006). Future analyses using data from MIDAS will include examining differences in susceptibility to PM by prior disease events, including MI.

Ambient PM has previously been associated with increased hospital admissions for COPD and asthma (Chen et al. 2004; Dominici et al. 2006; Medina-Ramon et al. 2006; Peel et al. 2005; Zanobetti and Schwartz 2003). COPD is associated with a greater propensity to hypoxia, reduced pulmonary reserve, and a generally heightened inflammatory state, which all may predispose to transmural infarcts.

Although several researchers have reported acute associations between PM and cardiovascular outcomes in panels of patients with diabetes or have reported greater changes in cardiorespiratory biomarkers among those with diabetes than those without diabetes (Dubowsky et al. 2006; Goldberg et al. 2001; Liu et al. 2007; O’Neill et al. 2005; Zanobetti and Schwartz 2002), we did not find greater susceptibility to PM among patients with diabetes. Similar to the findings of Zanobetti and Schwartz (2005), we found larger relative risks for patients with COPD than for those without this condition, but no difference in relative risk estimates for patients with diabetes versus those without diabetes.

Although our study has several strengths, including a large sample size, with subcategories of infarct and availability of continuous PM_2.5_ concentrations from seven monitoring stations during the study period, several limitations should be considered when interpreting our results. First, this study relies on administrative data only, and therefore some of the outcomes coded as an MI may not have been an MI, or those classified as transmural may have been miscoded as nontransmural, or vice versa. Specifically, we were limited to only ICD-9 codes to classify an MI as a transmural or nontransmural. However, a data audit was previously conducted that verified the accuracy of MI diagnoses as well as the accuracy of information in the MIDAS data set (Kostis et al. 2001). Thus, any outcome misclassification (MI vs. non-MI) was likely minimal. Further, any misclassification of transmural versus nontransmural was likely unrelated to ambient PM_2.5_ concentrations, resulting in nondifferential exposure misclassification and underestimates of relative risk.

Second, our data set included only those subjects who experienced an MI and were admitted to a hospital, and excluded MI patients who died before hospital admission. If PM does trigger transmural infarctions, which might have a higher out-of-hospital mortality rate, our data set would include a lower number of PM-triggered transmural infarctions than actually occurred during the study period. Therefore, our estimate of a 10% increase in the risk of a transmural infarction associated with each 10.8 μg/m^3^ increase in PM_2.5_ may be conservative.

Third, linking MIs to hourly and daily air pollution fluctuations requires minimizing misclassification of the estimate of MI onset time, so as to minimize bias. Although four previous studies (Peters et al. 2001; Peters et al. 2005; Pope et al. 2006; Sullivan J. et al. 2005) have used patients’ self-reported time of pain and symptom onset, this was not practical for our study, which relied on an administrative data set without personal interviews. Previous studies have reported median and mean delay times from symptom onset to ED arrival of 2.3–4.7 hr (Goldberg et al. 2002), with 44% of MI patients in Massachusetts arriving within 2 hr, and 78% arriving within < 6 hr (Goldberg et al. 2000). Thus, because there is often a delay of several hours from symptom onset to ED arrival, use of ED arrival hour instead of hour of symptom onset results in greater exposure misclassification and bias toward the null. Further, in our analysis, the mean PM_2.5_ concentrations in the 6, 12, and 24 hr before ED arrival were highly correlated (*r*-values = 0.83–0.95). Therefore, we did not examine associations with ambient PM_2.5_ concentrations < 24 hr before ED arrival, but instead focused on associations between infarctions and the ambient air pollution concentration in the 24 hr before ED arrival for that MI. Our finding of an association between increased 24-hr mean PM_2.5_ concentrations and transmural infarction needs to be replicated in a study that better estimates MI symptom onset time. This study would also allow a more proper investigation of the risk of a transmural infarction associated with PM_2.5_ concentrations < 24 hr before MI symptom onset than is possible in our analysis.

Fourth, because all of the 24 hr used to calculate the mean PM_2.5_ concentration in the 24 hr before ED arrival may not have been before the onset of MI symptoms, this again was a source of nondifferential exposure misclassification that may have resulted underestimates of relative risk. As reported by Lokken et al. (2009), this error in estimation of symptom onset time and the resulting relative risk–rate underestimation may be substantial in studies of acute cardiovascular and cerebrovascular events and short-term increases in PM. When comparing relative risk estimates based on symptom onset of stroke versus those based on hospital presentation for stroke, they observed an approximately 40% underestimation of the relative risk of stroke when using time of hospital presentation (Lokken et al. 2009).

Last, we assigned PM_2.5_ concentrations to all patients who lived < 10 km from a PM_2.5_ monitor, regardless of how close they lived to the monitor or how much time they spent at locations other than their residence. Because this error is not likely differential with respect to when a patient had the MI, it likely resulted in nondifferential exposure misclassification and underestimates of relative risk. In the two pollutant analyses adjusting for gaseous pollutant concentrations, gaseous pollutants may have greater degrees of spatial variability than does PM_2.5_ within this 10-km radius, resulting in residual confounding.

## Conclusions

We found increased risk of transmural infarctions, but not nontransmural infarctions, associated with each 10.8 μg/m^3^ increase in mean PM_2.5_ concentration in the 24 hr before ED arrival. Further, patients with COPD, but not those with diabetes, appeared particularly susceptible to effects of ambient particles. If our findings are confirmed, future investigations of PM and MI triggering should stratify by type of MI, with particular emphasis on transmural infarctions. Future work should also investigate mechanistic explanations for these findings.

## Figures and Tables

**Table 1:** Frequency and percentage of characteristics of study population (cases matched to PM_2.5_ monitors at ≤ 10 km distance): MIDAS study 2004–2006.

	Total MI (*n* = 5,864)	Nontransmural MI (*n* = 3,822)	Transmural MI (*n* = 1,563)
Characteristic	*n* (%)	*n* (%)	%
Age (years)			
18–44	376 (6)	214 (6)	137 (9)
45–54	842 (14)	454 (12)	326 (20)
55–64	1,245 (21)	736 (19)	404 (26)
65–74	1,187 (20)	796 (21)	297 (19)
75–84	1,431 (25)	1,027 (27)	277 (18)
≥ 85	783 (14)	595 (15)	122 (8)

Sex			
Male	3,296 (56)	2,048 (54)	980 (63)
Female	2,568 (44)	1,774 (46)	583 (37)

Race			
White	4,027 (69)	2,625 (69)	1,079 (69)
Black	901 (15)	623 (16)	180 (12)
Other	936 (16)	574 (15)	304 (19)

Year			
2004	1,634 (28)	1,066 (28)	452 (29)
2005	1,523 (26)	994 (26)	396 (25)
2006	2,707 (46)	1,762 (46)	715 (46)

Comorbidity			
Hypertension	3,658 (62)	2,506 (66)	864 (55)
Diabetes mellitus	1,761 (30)	1,207 (32)	423 (27)
Type I diabetes	136 (2)	103 (3)	22 (1)
Type II diabetes	1,625 (28)	1,104 (29)	401 (26)
COPD	839 (14)	608 (16)	164 (10)
Pneumonia	445 (8)	350 (9)	60 (4)
Heart disease	5,100 (87)	3,373 (88)	1,326 (85)
Ischemic heart disease	3,603 (61)	2,285 (60)	1,052 (67)
Congestive heart failure	1,878 (32)	1,427 (37)	299 (19)
Atrial fibrillation	971 (17)	723 (19)	174 (11)
Arrhythmia	1,793 (31)	1,177 (31)	466 (30)
Ventricular tachycardia	332 (6)	173 (5)	129 (8)

In-hospital procedure			
Angioplasty	786 (13)	393 (10)	350 (22)
CABG	288 (5)	202 (5)	60 (4)
Catheterization	3,178 (54)	1,908 (50)	1,028 (66)

**Table 2:** Distribution of daily mean PM_2.5_ concentrations at each monitoring station used.

Monitoring station	No. (%) of subjects matched to this monitor	Start and end dates of monitor during study period	No. of nonmissing days	PM_2.5_ concentration (μg/m^3^)				
				Minimum	25th	Median	75th	Maximum
Camden Lab	1,473 (25)	January 2004–December 2006	986	0.5	6.7	11.1	17.0	52.2
Elizabeth Lab	2,009 (34)	January 2004–December 2006	1,052	0	7.1	12.2	18.8	61.0
Flemington	35 (1)	January 2006–December 2006	259	0	3.9	7.6	12.8	38.4
Jersey City	661 (11)	January 2006–December 2006	336	1.4	6.0	9.7	18.2	46.9
Millville	148 (3)	January 2006–December 2006	348	0.6	6.6	10.3	16.1	48.4
New Brunswick	1,131 (19)	January 2004–December 2006	1,040	0.4	5.9	9.1	14.3	47.0
Rahway	407 (7)	January 2006–December 2006	246	5.1	9.0	12.3	18.3	48.8

**Table 3:** Pearson correlation coefficients for case and control period pollutant concentrations and apparent temperature.

Pollutant	PM_2.5_	NO_2_	SO_2_	CO	O_3_
PM_2.5_					
NO_2_	0.44				
SO_2_	0.44	0.56			
CO	0.33	0.63	0.42		
O_3_	0.19	−0.41	−0.32	−0.39	
Apparent temperature	0.35	−0.23	−0.29	−0.11	0.56

**Table 4:** Estimated risk of ED admission for MI (95% CI) associated with each 10.8-μg/m^3^ increase in moving average PM_2.5_ concentration, by infarction type.

Lag period	No. of infarctions	OR (95% CI)
All infarctions		
0–23	5,864	1.02 (0.98–1.07)
24–47	5,838	1.01 (0.97–1.06)
48–71	5,821	0.99 (0.95–1.04)
72–95	5,784	1.01 (0.97–1.05)
96–119	5,795	1.02 (0.98–1.07)
120–143	5,786	1.00 (0.96–1.05)
144–167	5,770	1.00 (0.96–1.04)
Transmural infarctions		
0–23	1,563	1.10 (1.01–1.20)
24–47	1,560	1.02 (0.93–1.11)
48–71	1,548	1.04 (0.96–1.13)
72–95	1,544	1.03 (0.95–1.12)
96–119	1,554	1.05 (0.97–1.13)
120–143	1,558	1.04 (0.97–1.13)
144–167	1,551	0.97 (0.90–1.05)
Nontransmural infarctions		
0–23	3,822	0.99 (0.94–1.05)
24–47	3,803	1.00 (0.94–1.06)
48–71	3,805	0.96 (0.91–1.01)
72–95	3,771	0.98 (0.93–1.04)
96–119	3,773	1.01 (0.96–1.06)
120–143	3,766	0.99 (0.94–1.04)
144–167	3,758	1.00 (0.95–1.05)

Each estimate of the risk of MI associated with each IQR increase in lagged PM_2.5_ concentration was modeled separately, adjusting for apparent temperature during that same lag period.

**Table 5:** Risk of ED admission for transmural infarction (and 95% CI) associated with each 10.8-μg/m^3^ increase in the mean PM_2.5_ concentration in the previous 24 hr.

Characteristic	No. of infarctions	OR (95% CI)	*p*-Value for interaction
COPD			
Yes	164	1.32 (1.05–1.66)	0.10
No	1,399	1.07 (0.98–1.18)	

Diabetes			
Yes	423	1.06 (0.90–1.23	0.54
No	1,140	1.11 (1.01–1.23)	

Age (years)			
< 65	867	1.18 (1.05–1.31)	0.05
≥ 65	696	1.01 (0.89–1.14)	

Sex			
Male	980	1.12 (1.01–1.24)	0.54
Female	583	1.07 (0.94–1.21)	

Race			
White	1,079	1.11 (1.01–1.23)	—
Black	180	1.14 (0.93–1.41)	0.79^a^
Other	304	1.02 (0.84–1.23)	0.40^b^

Season			
Winter	345	1.08 (0.90–1.30)	0.90
Summer	404	1.09 (0.94–1.26)	

Each estimate of the risk of MI associated with each IQR increase in lagged PM_2.5_ concentration was modeled separately, adjusting for apparent temperature during that same lag period.

a Comparing black with white.

b Comparing other with white.

**Table 6:** Risk of transmural infarction (and 95% CI) associated with each IQR increase in mean pollutant concentration in the previous 24 hr.

Pollutant	Model type	No. of infarctions	OR (95% CI)
PM_2.5_	Single pollutant	1,262	1.10 (1.00–1.21)
NO_2_	Single pollutant		1.11 (0.97–1.25)
PM_2.5_	Two pollutant		1.08 (0.96–1.22)
NO_2_	Two pollutant		1.04 (0.88–1.22)

PM_2.5_	Single pollutant	1,183	1.08 (0.98–1.19)
CO	Single pollutant		1.02 (0.93–1.12)
PM_2.5_	Two pollutant		1.10 (0.98–1.22)
CO	Two pollutant		0.97 (0.87–1.08)

PM_2.5_	Single pollutant	1,238	1.08 (0.98–1.18)
SO_2_	Single pollutant		1.02 (0.93–1.11)
PM_2.5_	Two pollutant		1.10 (0.98–1.24)
SO_2_	Two pollutant		0.96 (0.86–1.08)

PM_2.5_	Single pollutant	1,003	1.08 (0.97–1.21)
O_3_	Single pollutant		0.95 (0.81–1.12)
PM_2.5_	Two pollutant		1.08 (0.97–1.21)
O_3_	Two pollutant		0.95 (0.81–1.11)

IQR = 10.8 μg/m^3^ for PM_2.5_, 16 ppb for NO_2_, 0.35 ppm for CO, 4.1 ppb for SO_2_, and 18 ppb for O_3_.
